# A Novel Type II NAD^+^-Specific Isocitrate Dehydrogenase from the Marine Bacterium *Congregibacter litoralis* KT71

**DOI:** 10.1371/journal.pone.0125229

**Published:** 2015-05-05

**Authors:** Ming-Cai Wu, Chang-Qing Tian, Hong-Mei Cheng, Lei Xu, Peng Wang, Guo-Ping Zhu

**Affiliations:** 1 Institute of Molecular Biology and Biotechnology, Anhui Normal University, No. 1 Beijing East Road, Wuhu, 241000, Anhui, China; 2 Anhui Province Key Laboratory of Active Biological Macro-molecules, Wannan Medical College, No. 22 Wenchang West Road, Wuhu, 241002, Anhui, China; University of Freiburg, GERMANY

## Abstract

In most living organisms, isocitrate dehydrogenases (IDHs) convert isocitrate into ɑ-ketoglutarate (ɑ-KG). Phylogenetic analyses divide the IDH protein family into two subgroups: types I and II. Based on cofactor usage, IDHs are either NAD^+^-specific (NAD-IDH) or NADP^+^-specific (NADP-IDH); NADP-IDH evolved from NAD-IDH. Type I IDHs include NAD-IDHs and NADP-IDHs; however, no type II NAD-IDHs have been reported to date. This study reports a novel type II NAD-IDH from the marine bacterium *Congregibacter litoralis* KT71 (ClIDH, GenBank accession no. EAQ96042). His-tagged recombinant ClIDH was produced in *Escherichia coli* and purified; the recombinant enzyme was NAD^+^-specific and showed no detectable activity with NADP^+^. The *K*_m_ values of the enzyme for NAD^+^ were 262.6±7.4 μM or 309.1±11.2 μM with Mg^2+^ or Mn^2+^ as the divalent cation, respectively. The coenzyme specificity of a ClIDH Asp487Arg/Leu488His mutant was altered, and the preference of the mutant for NADP^+^ was approximately 24-fold higher than that for NAD^+^, suggesting that ClIDH is an NAD^+^-specific ancestral enzyme in the type II IDH subgroup. Gel filtration and analytical ultracentrifugation analyses revealed the homohexameric structure of ClIDH, which is the first IDH hexamer discovered thus far. A 163-amino acid segment of CIIDH is essential to maintain its polymerization structure and activity, as a truncated version lacking this region forms a non-functional monomer. ClIDH was dependent on divalent cations, the most effective being Mn^2+^. The maximal activity of purified recombinant ClIDH was achieved at 35°C and pH 7.5, and a heat inactivation experiment showed that a 20-min incubation at 33°C caused a 50% loss of ClIDH activity. The discovery of a NAD^+^-specific, type II IDH fills a gap in the current classification of IDHs, and sheds light on the evolution of type II IDHs.

## Introduction

Isocitrate dehydrogenase (IDH) belongs to a large, ubiquitous, and very ancient protein family whose members play central roles in energy metabolism, amino acid biosynthesis, and vitamin production. IDH catalyzes the oxidative NAD(P)^+^-dependent dehydrogenation and decarboxylation of isocitrate to α-ketoglutarate (α-KG) and CO_2_. IDHs can be divided into two major groups according to coenzyme specificity: NAD^+^-specific IDHs (EC 1.1.1.41, NAD-IDH) and NADP^+^-specific IDHs (EC 1.1.1.42, NADP-IDH). Eukaryotic NAD-IDHs are exclusively localized in the mitochondria, producing NADH for energy metabolism, whereas eukaryotic NADP-IDHs are found in different cellular compartments and participate in diverse cellular processes. Human NADP-IDHs have recently been reported to be involved in tumorigenesis [1–3]. Both the Arg132 mutation of cytosolic NADP-IDH and the Arg172 mutation of mitochondrial NADP-IDH can impair the oxidative activity of IDH, and they confer a new function of reducing α-ketoglutarate to 2-hydroxyglutarate to the mutant enzymes [4, 5]. The resulting accumulation of 2-hydroxyglutarate can induce the formation and malignant progression of tumors [6–8].

**Fig 1:**
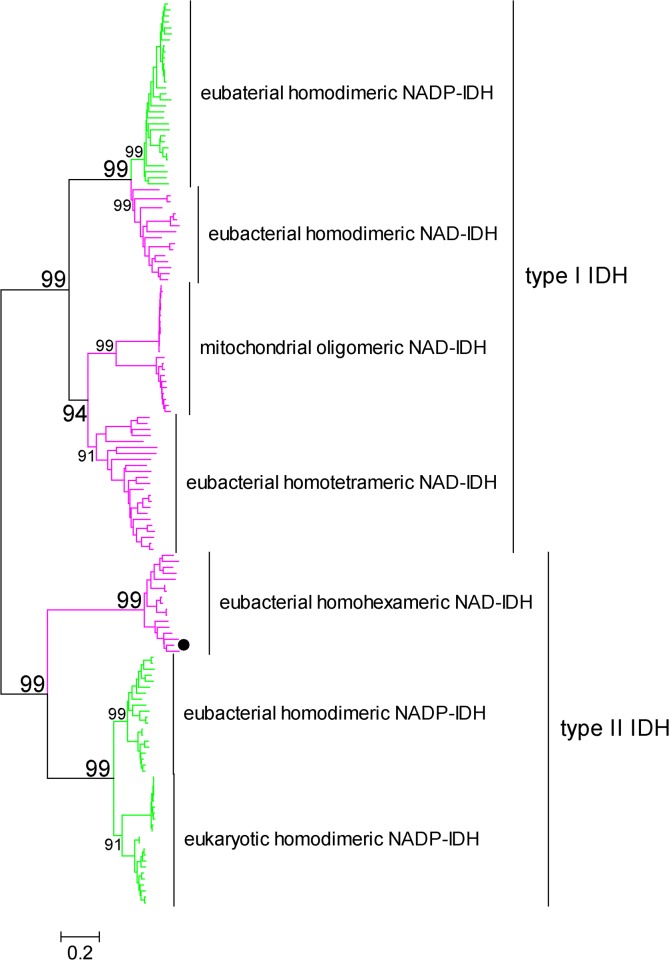
Evolutionary relationships between 151 IDHs from different organisms. Green branches represent NADP-IDHs, and pink branches represent NAD-IDHs. The evolutionary history was inferred using the neighbor-joining method [35]. The bootstrap consensus tree inferred from 500 replicates represents the evolutionary history of the analyzed taxa [36]. Branches corresponding to partitions that are reproduced in less than 50% of the bootstrap replicates are collapsed. The percentages of replicate trees in which the associated taxa clustered together in the bootstrap test are shown next to the branches [36]. The tree is drawn to scale, with branch lengths presented in the same units as the evolutionary distances that were used to infer-he phylogenetic tree. The evolutionary distances were computed using the Poisson correction method [37] and are shown in units of ‘number of amino acid substitutions per site’. All positions containing gaps and missing data were eliminated from the dataset (complete deletion option). A total of 255 positions were in the final dataset. Phylogenetic analyses were conducted using MEGA4 [15].

Although all IDHs catalyze identical reactions, i.e., the oxidation of isocitrate, their amino acid compositions are highly diverse. The IDH protein family can be divided into two main subgroups, type I and type II IDHs, according to sequence-based phylogenetic analysis [9, 10]. Four types of IDHs constitute the type I subfamily: eubacterial homodimeric NADP^+^-specific IDHs, eubacterial homodimeric NAD^+^-specific IDHs, eubacterial homotetrameric NAD^+^-specific IDHs and mitochondrial oligomeric NAD^+^-specific IDHs. Both NAD^+^-specific and NADP^+^-specific IDHs can be found in the type I subfamily. However, all type II IDHs that have been reported to date are NADP^+^-specific, including eubacterial homodimeric NADP^+^-specific IDHs and eukaryotic homodimeric NADP^+^-specific IDHs (Fig 1).

We have previously shown that prokaryotic NAD^+^-specific IDHs are ancestors of the IDH protein family and that the use of NADP^+^ by IDH arose at approximately the same time that eukaryotic mitochondria first appeared, approximately 3.5 billion years ago [10]. The switch in cofactor specificity from NAD^+^ to NADP^+^ was an ancient adaptive event facilitating organism survival on acetate or other two-carbon, energy-poor resources by generating NADPH, which has more reducing power. The evolutionary pathway from NAD-IDH to NADP-IDH in the type I IDH subfamily has been clearly elucidated; however, the possible existence of a similar evolutionary scenario in the type II IDH subfamily has never been explored because no type II NAD^+^-specific IDHs (the potential ancestors) have been discovered so far. Therefore, the identification of type II NAD-IDHs will be particularly significant for supporting the hypothesis that the type II IDH subfamily evolved the same adaptive strategy of switching cofactor dependence to improve organism survival. Additionally, the discovery of type II NAD-IDHs could help to expand the current classification of the IDH family.

The present study describes a novel type II, NAD^+^-specific IDH from the marine bacterium *Congregibacter litoralis* KT71 (ClIDH, GenBank accession no. EAQ96042). ClIDH was heterologously produced as a fusion protein in *Escherichia coli*, and its biochemical properties were characterized in detail. The coenzyme specificity of ClIDH can be converted from NAD^+^ to NADP^+^ via site-directed mutagenesis, suggesting that ClIDH represents an ancient member of the type II IDHs. Our results also reveal ClIDH to be the first homohexameric IDH reported to date. A sequence segment in ClIDH composed of 163 amino acid residues was shown to be necessary for the hexamerization of this enzyme, as a truncated version of ClIDH lacking this region was monomeric. Collectively, our data suggest that ClIDH is a novel IDH family member, which may provide new insights into the evolution of type II IDHs.

## Materials and Methods

### Microbial strains and growth conditions

The *E*. *coli* DH5α and Rosetta (DE3) strains were preserved in our laboratory. The *C*. *litoralis* strain KT71 was purchased from the NITE Biological Resource Center (NBRC, Japan) and grown on the complex SYPG medium described at http://www.nbrc.nite.go.jp/NBRC2/NBRCMediumDetailServlet?NO=1070. In brief, the medium contained 35.0 g sea salts (Sigma, USA), 0.5 g yeast extract, 0.25 g trypticase peptone (Sigma, USA), 0.5 g sodium carbonate, 0.1 g sodium glutamate, 0.1 g NH_4_Cl, 0.05 g KH_2_PO_4_, 1.0 mL Wolfe’s mineral elixir and 10 mL vitamin solution in 1 L demineralized water. First, the medium without carbonate and vitamins was prepared and sparged with an N_2_/CO_2_ (80/20) gas mixture until it was anoxic and saturated with CO_2_ (for at least 30 min). Then, the medium was sterilized by filtration and dispensed into sterile (autoclaved) vials suitable for anaerobic cultures filled with an N_2_/CO_2_ (80/20) gas mixture. The vials were sealed with butyl rubber septa (to prevent free exchange of oxygen with the external atmosphere) and filled with the medium at 50% of their volume. Finally, filter-sterilized carbonate (from 5% (w/v) stock solution) and vitamins solution were anaerobized and added to the vials, and pH of the medium was adjusted to 7.4–7.8 with sterile carbonate. After cultivation of *C*. *litoralis* at 28°C for 7 days, the cells were harvested by centrifugation (2,000 g, 4°C, 15 min) and disrupted by sonication. This was followed by a centrifugation step (12,000 g, 4°C, 20 min), and the supernatant was used for enzymatic analysis.

### Plasmid construction

Plasmid DNA was extracted using the Wizard purification kit (Promega, USA), and PrimeSTAR HS DNA polymerase was obtained from TaKaRa (Dalian, China). Restriction enzymes and protein molecular weight standards were purchased from Fermentas (Shanghai, China). Genomic DNA from *C*. *litoralis* KT71 was kindly provided by Dr. Bernhard M. Fuchs from the Max Planck Institute for Marine Microbiology, Germany. One pair of specific primers was designed according to the *ClIDH* gene sequence of *C*. *litoralis* KT71 (GenBank accession no. AAOA02000001, 1256088–1257830). The sense primer was 5’-GGAATTCCATATGAGCAACAAAATCAAGGTAGAGAAGC-3’ (*Nde*I site underlined), and the antisense primer was 5’-ATAAGAATGCGGCCGCTTATGAGGCGTTGAGGTTTGCTTCA -3’ (*Not*I site underlined). PCR amplification was performed for 35 cycles with the following steps: 30 s at 94°C, 30 s at 55°C, and 1.5 min at 72°C. The PCR products were digested using *Nde*I and *Not*I and ligated into pET-28b(+) to create the recombinant plasmid pET-*ClIDH*, which contained a 6×His tag coding sequence upstream of the multiple cloning site. The ClIDH gene was confirmed via DNA sequencing.

### Site-directed mutagenesis and construction of a truncated enzyme

Two point mutations were introduced into ClIDH by overlap extension PCR-based site-directed mutagenesis to produce the two amino acid substitutions Asp487Arg and Leu488His. The following oligonucleotides were used to generate the mutant enzyme: forward primer, 5’-CACGGCACGGCCCACCGTCATTATCTGCGGTATCTC-3’, and reverse primer, 5’-GAGATACCGCAGATAATGACGGTGGGCCGTGCCGTG-3’. The underlined codons represent mutated sequences. A truncated ClIDH (shortened by 163 amino acid residues, from Asn272 to Arg314 and Gly319 to Trp438) was produced using the following oligonucleotides: forward primer, 5’-CTGGGCGGGATACAGGCCACCGCCGGCGCTTGACACGATCCGCGCTGCCTGGGC-3’, and reverse primer, 5’-CAGCGCGGATCGTGTCAAGCGCCGGCGGTGGCCTGTATCCCGCCCAGAACCTC-3’. The mutated gene fragments were then inserted into the pET-28b(+) expression vector, and their sequences were confirmed through DNA sequencing.

### Expression and purification of ClIDH

*E*. *coli* Rosetta (DE3) cells harboring the expression plasmids were cultured overnight in LB medium containing 30 μg/mL kanamycin and 30 μg/mL chloramphenicol at 37°C. The cells were then inoculated into 50 mL of fresh LB media with the same antibiotic and grown until mid-log phase or until a cell density of A_600nm_ = 0.4. Isopropyl-1-thio-β-D-galactopyranoside (IPTG) was added to the culture at a final concentration of 0.5 mM, followed by overnight culturing at 20°C. The cells were then harvested via centrifugation at 2,000 g at 4°C for 15 min, resuspended in a lysis buffer containing 50 mM Tris-HCl (pH 7.5) and 500 mM NaCl, and disrupted by sonication. The insoluble debris was removed through centrifugation at 12,000 g for 20 min at 4°C. Recombinant 6xHis-tagged ClIDH was purified using BD TALON Metal Affinity Resin (Clontech, USA) according to the manufacturer’s instructions.

### SDS-PAGE and western blotting

The purity of the recombinant proteins was evaluated on 12% SDS-PAGE gels. For the western blot analysis, protein samples (25 μg each) were separated via SDS-PAGE and transferred to nitrocellulose membranes (Amersham Biosciences, Germany) through electroblotting. The membranes were blocked for 1 h at room temperature in TBST (50 mM Tris-HCl (pH 7.5), 150 mM NaCl, 0.2% Tween-20) containing 5% nonfat milk and probed with an anti-His-tag polyclonal antibody (Cell Signaling Technology, USA, 1:2000) and then with an alkaline phosphatase-conjugated anti-rabbit IgG secondary antibody (Promega, USA, 1:3000). The blots were washed three times with 20 mL TBST at room temperature for 5 min on a rolling device. The membrane was then incubated at room temperature for 10 min on a rolling device with sufficient alkaline phosphatase substrate to cover the blot (3 mL). The chemiluminescence signals from specific antibody-antigen reactions were visualized by exposing the blots to X-ray film for 15 min in a dark room.

### Enzyme assays

The enzymatic activities of the wild-type and mutant ClIDH proteins were assayed using a method developed by Cvitkovitch et al. [11], with some modifications. The assays were carried out at 25°C in 1-mL cuvettes (the light path was 1 cm) containing 35 mM Tris-HCl buffer (pH 7.5) with 2 mM MgCl_2_ or MnCl_2_, 1.5 mM DL-isocitrate, and 1.0 mM NAD^+^ or 2.0 mM NADP^+^. NADH or NADPH production was monitored at 340 nm using a thermostated Cary 300 UV-Vis spectrophotometer (Varian, USA) with a molar extinction coefficient of 6.22 mM^-1^ cm^-1^. One unit of enzyme activity represented the reduction of 1 μmol of NAD^+^ or NADP^+^ per minute. Protein concentrations were determined using the Bio-Rad protein assay kit (Bio-Rad, USA), with bovine serum albumin as a standard.

### Characterization of recombinant ClIDH

Using the standard enzyme assay described above, the cofactor concentration was keeped at 1 mM, while the isocitrate concentration was varied, to measure the *K*_m_ value for isocitrate. Conversely, the isocitrate concentration was keeped at 1.5 mM, while the cofactor concentration was varied, to measure the *K*_m_ and *V*_max_ values for NAD^+^. The apparent *V*_max_ and *K*_m_ values were calculated via nonlinear regression using the Prism 5.0 software (Prism, USA). All kinetic parameters were obtained from at least three measurements.

The effects of pH and temperature on activity of recombinant ClIDH were examined using the enzyme assay described above. The activity of the purified recombinant ClIDH was measured in 35 mM Tris-HCl buffer (between pH 7.0 and 9.0) containing Mn^2+^or Mg^2+^, and the optimum temperature was determined within the range of 20–45°C. The half-life of recombinant ClIDH was determined using the heat inactivation method as follows: enzyme aliquots were incubated at 25–38°C for 20 min and then immediately cooled on ice, and the residual activity was measured.

The effects of different metal ions, including 2 mM monovalent ions (K^+^, Li^+^, Na^+^ and Rb^+^) and divalent ions (Ca^2+^, Co^2+^, Cu^2+^, Mg^2+^, Mn^2+^, Ni^2+^ and Zn^2+^), on the activity of recombinant ClIDH were determined using the same enzyme assay described above.

### Gel filtration chromatography

The molecular mass of recombinant ClIDH was detected via gel filtration chromatography on a 10/300 Superdex 200 column (Amersham Biosciences, Germany) equilibrated with 0.05 M potassium phosphate buffer (pH 7.0) containing 0.15 M NaCl and 0.01% sodium azide. The protein standards used for calibration were ovalbumin (45 kDa), conalbumin (75 kDa), aldolase (158 kDa), ferritin (440 kDa) and thyroglobulin (669 kDa).

### Sedimentation velocity

Analytical ultracentrifugation (AUC) experiments were performed with a Beckman XL-A analytical ultracentrifuge equipped with a UV scanning system and using a 4-hole An-60 Ti rotor and a test time interval of 3 min. The reference loading volume was 410 μL, and the sample loading volume was 400 μL. In a typical experiment, 200 absorbance profiles were recorded at 20°C and 116,000 g. The test mode was based on the sedimentation velocity, and a continuous c(s) distribution was applied in the analysis mode. The derivative profiles were used to calculate the experimental sedimentation coefficient (*s*_exp_). The data were also analyzed using the Svedberg program [12]. The Sednterp program, which was developed by Haynes DB, Laue T, and Philo J and can be found at http://bbri.org/RASMB/rasmb.html was used to calculate the partial specific volume (*v*_2_), solvent density (ρ), and viscosity (η). The value of the partial specific volume was 0.7367 for ClIDH, and the corrected coefficient, *s*_20,w_, was calculated using the following equation: *s*_20,w_ = *s*_exp_ (η/η_w,20_) (1-ρ_w,20_· *v*_2_)/ (1-ρ·*v*_2_).

### Sequence alignment and construction of a phylogenetic tree

The X-ray structure of *Mycobacterium tuberculosis* NADP-IDH1 (MtIDH, 4HCX) was downloaded from the PDB database (http://www.rcsb.org/pdb/). A homology model for ClIDH was generated using the SWISS-MODEL server (http://swissmodel.expasy.org). Amino acid sequence alignment was conducted using the ClustalX program (ftp://ftp.ebi.ac.uk/pub/software/clustalw2), and a structure-based sequence alignment was subsequently generated using the ESPript 2.2 web tool (http://espript.ibcp.fr/ESPript/ESPript/) [13, 14].

A total of 207 IDH sequences from diverse sources were downloaded from GenBank via the NCBI website (http://www.ncbi.nlm.nih.gov/). A bootstrapped neighbor-joining tree was constructed using MEGA 4 (http://www.megasoftware.net/) and was based on sequence alignment with ClustalX [13, 15].

## Results and Discussion

### Novel NAD^+^-specific ClIDH represents an ancestral type II IDH

**Fig 2:**
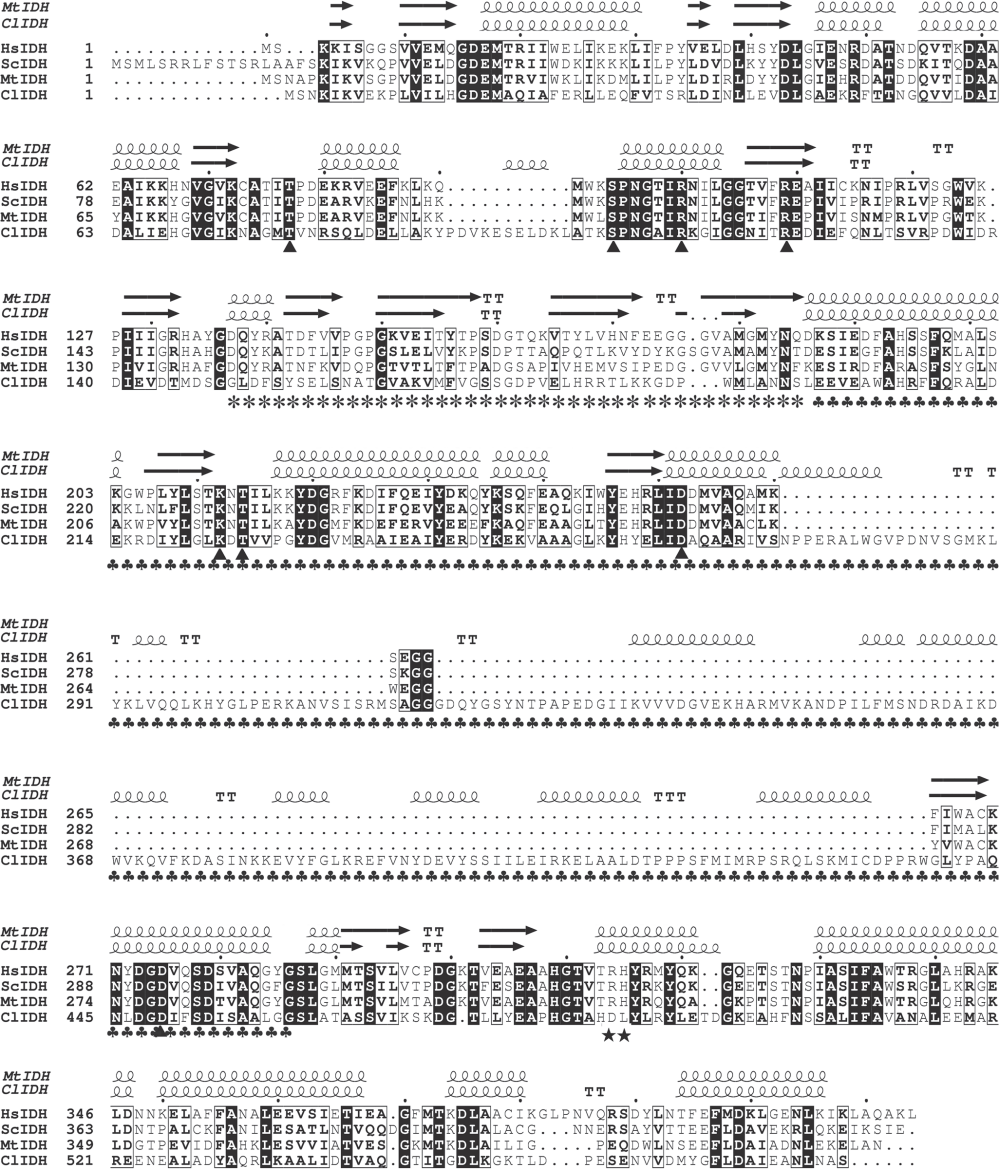
Structure-based protein sequence alignment of ClIDH with three typical type II homodimeric IDHs. The three typical type II homodimeric IDHs were from *Homo sapiens* (human) (HsIDH, GenBank accession no. NP_005887.2), *Saccharomyces cerevisiae* (yeast) (ScIDH, GenBank accession no. P21954) and *Mycobacterium tuberculosis* (MtIDH, GenBank accession no. WP_003904258.1). The high-resolution structure of MtIDH (PDB ID, 4HCX) was downloaded from the PDB database. The ClIDH homology model was generated using the SWISS-MODEL server. Invariant residues are highlighted with shaded blue boxes, and conserved residues are highlighted with open blue boxes. The conserved residues that are involved in cofactor binding (★) and substrate binding (▲) are indicated. The clasp region is represented by (*), and the small domain is represented by (♣). The figure was generated using ESPript 2.2 [14].

Although all known IDHs in the type II subfamily are NADP^+^-dependent [16–22], the large number of IDH sequences that are currently available in GenBank has made it possible to identify the ancestral, NAD^+^-dependent type II IDHs. When examining the relevant protein sequences in the database, the IDH from *C*. *litoralis* KT71 (ClIDH) drew our attention due to its special sequence features. ClIDH shares higher sequence identity with type II IDHs (~30%) than with type I IDHs (~20%). A secondary structure-based sequence alignment between ClIDH and three well-characterized, type II NADP-IDHs from *Homo sapiens* [23], *Saccharomyces cerevisiae* [24] and *M*. *tuberculosis* [25] was conducted (Fig 2). ClIDH showed remarkable structural similarity to the typical type II IDHs, and the residues that are involved in substrate binding are completely conserved among these homologs (Fig 2). However, two crucial amino acid residues involved in coenzyme binding of NADP-IDHs, Arg and His, were replaced by Asp and Leu in ClIDH (Fig 2). Because the existence of Asp at the cofactor-binding site of IDH can be considered to be a hallmark of NAD^+^ specificity [9,26], we postulated that ClIDH might be a novel, NAD^+^-specific type II IDH. This NAD^+^ specificity was subsequently confirmed via kinetic analysis.

ClIDH is not the only NAD^+^-dependent type II IDH in the database. BLAST analysis using ClIDH as the query revealed that there are 38 putative IDHs of this type in the GenBank database. These IDHs share very high sequence identities (67% to 89%) with ClIDH, and all of them originate from marine microorganisms, such as *Luminiphilus syltensis*, *Halomonas anticariensis*, *Marinobacter manganoxydans* and *Marinobacter adhaerens*. Sequence alignment showed that the NAD^+^-discriminative Asp is present at the coenzyme binding sites of all 38 IDHs, suggesting that all of these IDHs are NAD^+^-specific (S1 Fig). Furthermore, these 38 IDHs were clustered together in the phylogenetic tree, as expected (Fig 1). Taken together, this novel, NAD^+^-dependent IDH protein subfamily, represented by ClIDH, can be regarded as the ancestor of the modern NADP^+^-specific, type II IDHs according to the evolutionary mechanism of IDH coenzyme specificity described in our previous study [10].

### Expression and purification of recombinant ClIDH

**Fig 3:**
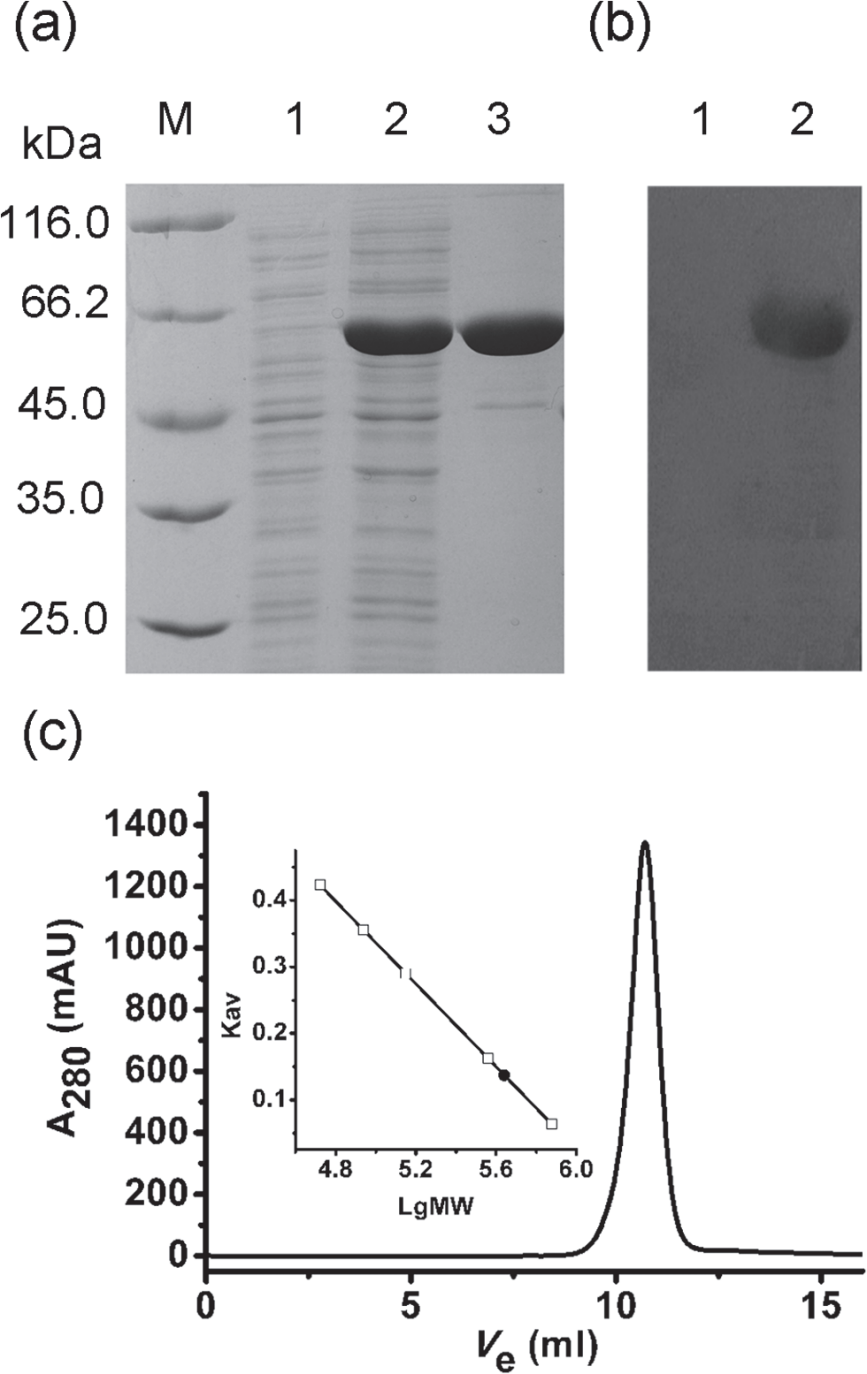
Purification of recombinant ClIDH. (a) Protein purity was assessed via 12% SDS-PAGE. M, protein marker; lane 1, crude extracts of cells harboring pET-28b(+) after induction with IPTG; lane 2, crude extracts of cells harboring the recombinant plasmid after induction with IPTG; lane 3, purified protein. (b) Western blot analysis using the anti-6×His antibody as a probe. Lane 1, negative control, crude extracts of cells harboring pET-28b(+) with IPTG induction; lane 2, purified protein. (c) Molecular mass determination via gel filtration chromatography. The V_e_ of recombinant ClIDH was 10.7 mL.

The ClIDH gene is 1,743 bp in length and encodes a protein of 580 amino acids. The monomeric molecular weight (MW) of recombinant ClIDH, together with the 6×His-tag, was found to be approximately 64 kDa (Fig 3A), which coincided well with the theoretical calculation (64.4 kDa) performed using the ProtParam tool on the ExPASy server (http://www.au.expasy.org/tools/protparam.html). This MW is higher than that of the typical type II homodimeric NADP-IDHs, which is approximately 42 kDa. The excess 20 kDa of mass is mainly attributed to a stretch of 163 amino acid residues (Asn272-Met314 and Gly319-Trp438) located in the middle of the ClIDH sequence, which is absent in the typical type II homodimeric NADP-IDHs (Fig 2). Western blot analysis using an anti-6×His antibody verified the identity of the purified enzyme. One specific protein band was revealed, demonstrating that the recombinant ClIDH was successfully expressed and purified (Fig 3B).

### ClIDH forms a homohexamer in solution

**Fig 4:**
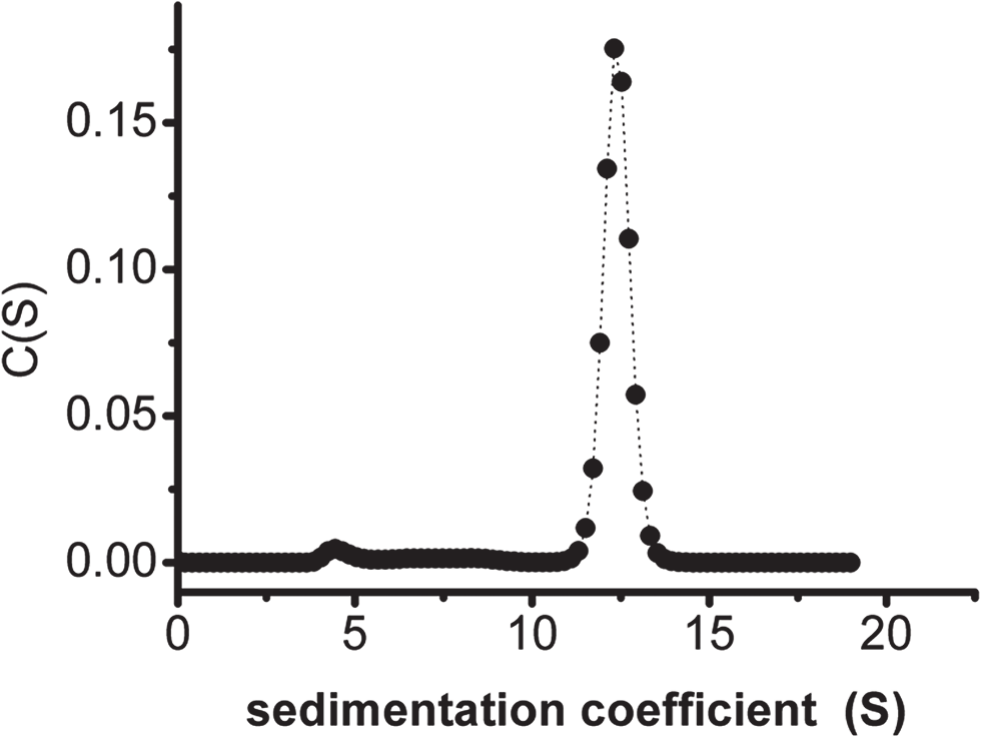
Histogram plot showing the sedimentation coefficient distribution of ClIDH at 20°C.

Gel filtration chromatography was conducted to determine the oligomerization status of ClIDH in solution. Recombinant ClIDH was eluted as a symmetrical peak between aldolase and ferritin, revealing a molecular mass of approximately 390 kDa (Fig 3C). This result strongly suggested that the recombinant ClIDH formed a homohexamer in solution. Dimeric fraction of ClIDH was not detected. The sedimentation velocity of ClIDH was further analyzed to determine its association state with greater accuracy. A single analytical centrifugation run was recorded and used protein extraction with more than 90% purity via gel filtration chromatography. The distribution of the sedimentation coefficient showed a major peak, with an *s*_20,W_ of 12 S (Fig 4). The AUC data were analyzed using SEDFIT 12.44. The calculated molecular mass of ClIDH was approximately 350 kDa, a value that was close to both the expected theoretical value and the value that was experimentally determined through gel filtration (390 kDa), which is indicative of a homohexamer.

ClIDH is the first homohexameric member of the IDH family that has been reported to date. Most IDHs, regardless of their coenzyme dependency and organismal origin, have been shown to be homodimeric or monomeric [21]. In addition to these two common oligomer structures, a small number of homotetrameric IDHs have been found in certain bacteria, such as *Thermotoga maritima* and *Methylococcus capsulatus* (Fig 1) [20, 27]. These homotetrameric IDHs, together with the homohexameric ClIDH and its counterparts identified in this study, demonstrate that higher-order IDH homooligomers certainly exist in nature.

### The additional 163 amino acids are essential to maintain ClIDH polymerization and activity

The structure of a protein is determined by its primary sequence. Therefore, we reasoned that the homohexameric status of ClIDH in solution should be explained by its amino acid sequence. ClIDH was aligned with three typical type II, homodimeric NADP-IDHs (Fig 2). Although the sequence identities between ClIDH and these three IDHs were only approximately 25%, the most distinguishing difference was the extra 163 amino acid residues (Asn272-Met314 and Gly319-Trp438) harbored by ClIDH (Fig 2). If these 163 amino acids were not taken into account, the sequence identity between ClIDH and the three homodimeric NADP-IDH was much higher (35%).

The dimerization of human cytosolic NADP-IDH (HsIDH in Fig 2) is accomplished via interactions between the two clasp regions and the two small domains of each subunit [23]. The putative position and length of the clasp region in ClIDH are homologous to the corresponding region in HsIDH, suggesting that this region may be involved in the interaction among the clasp regions of ClIDH (Fig 2). Furthermore, sequence alignment showed that an additional 163 amino acids in ClIDH are inserted into the region corresponding to the small domain of HsIDH (Fig 2), implying that this insertion may play essential role for the hexamerization of ClIDH.

**Fig 5:**
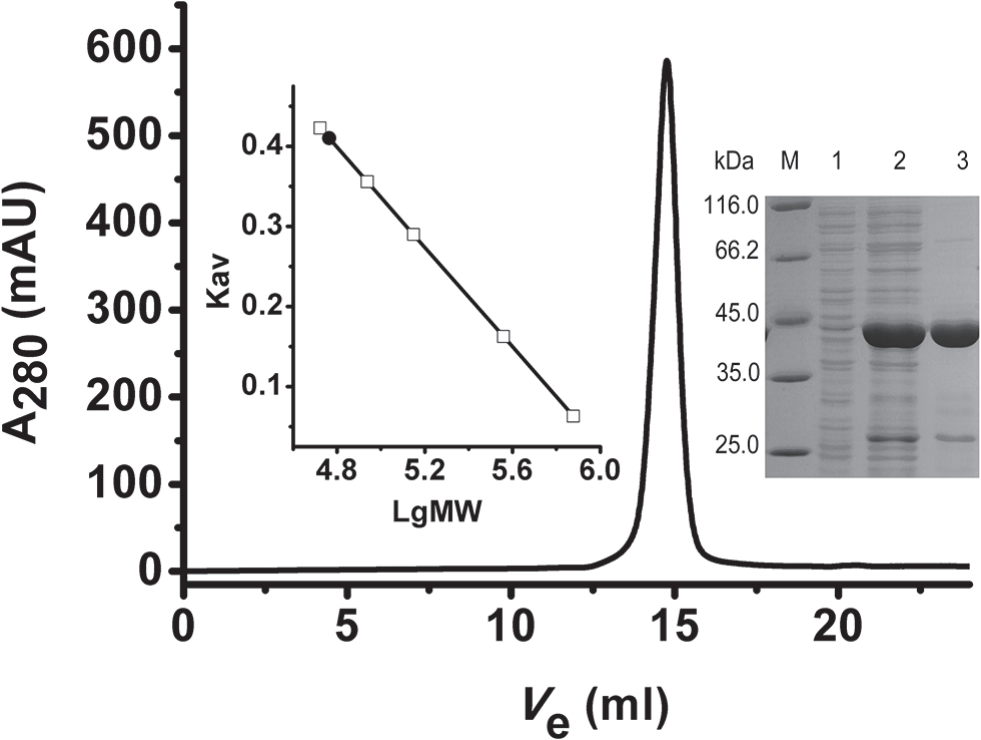
Molecular mass determination of truncated ClIDH via gel filtration chromatography. The V_e_ of the truncated ClIDH is 14.77 mL.

To determine whether this extra sequence segment is essential for ClIDH to maintain polymerization and activity, a fusion PCR-based method was used to construct a truncated ClIDH variant lacking the 163 amino acids. The truncated ClIDH was successfully expressed in *E*. *coli*, and its oligomeric state was subsequently determined. SDS-PAGE analysis revealed that the molecular mass of the truncated ClIDH protein was 45 kDa, which was consistent with the calculated mass (45.7 kDa) (Fig 5). The native molecular weight of the truncated ClIDH was estimated to be approximately 50 kDa via gel filtration chromatography, which is suggestive of a monomeric structure (Fig 5). This result suggested that the additional 163-amino acid sequence is important for maintaining the stable hexamer structure of ClIDH. Furthermore, truncated ClIDH showed no activity towards NAD^+^ or NADP^+^ according to the standard enzyme assay, demonstrating that this 163-amino acid segment is essential for the catalysis.

### Enzyme activity and kinetic characterization

The specific activities of recombinant ClIDH for NAD^+^ in the presence of Mn^2+^ and Mg^2+^ were determined to be 61 U/mg and 29 U/mg, respectively. Kinetic characterization of recombinant ClIDH showed that its apparent *K*_m_ for NAD^+^ was 262.6±7.4 μM or 309.1±11.2 μM in the presence of Mg^2+^ or Mn^2+^, respectively (Table 1). No NADP^+^-associated activity was observed when NAD^+^ was substituted with NADP^+^ at concentrations up to 2 mM, demonstrating that recombinant ClIDH was NAD^+^-specific. These *K*_m_ values are similar to those of the homodimeric NAD-IDHs of *Zymomonas mobilis* (312 μM) [28] and *Streptococcus suis* (233 μM) [26], whereas they are higher than the *K*_m_ values of the homotetrameric NAD-IDH from *M*. *capsulatus* (122 μM) [27] and of the homodimeric NAD-IDH from *Acidithiobacillus thiooxidans* (180 μM) [29] and lower than the *K*_m_ of the NAD-IDH from *Hydrogenobacter thermophilus* (357 μM) [30].

**Table 1:** Kinetic parameters of wild-type and mutant ClIDH.

Enzyme	NAD^+^			NADP^+^		
	*K*_m_ (μM)	*k*_cat_ (s^-1^)	*k*_cat_/*K*_m_ (μM^-1^ s^-1^)	*K*_m_ (μM)	*k*_cat_ (s^-1^)	*k*_cat_/*K*_m_ (μM^-1^ s^-1^)
ClIDH (Mg^2+^)	262.6±7.4	36.7	0.14	−	−	−
ClIDH (Mn^2+^)	309.1±11.2	84.7	0.27	−	−	−
D487R/L488H (Mn^2+^)	663.8±48.6	9.7	0.015	156.7±4.8	55.1	0.35

The specific activity of IDH in crude extracts of *C*. *litoralis* KT71 was 6.4 and 6.1 U/mg in the presence of NAD^+^ and NADP^+^, respectively. Considering that the purified 6xHis-tagged ClIDH had no detectable activity towards NADP^+^, we deduced that the NADP^+^-dependent IDH activity originated from another IDH in *C*. *litoralis* KT71 (GenBank accession no. EAQ99191) which is likely to be an NADP^+^-specific enzyme, similar to *E*. *coli* IDH, due to its highly conserved NADP^+^-binding sites (S2 Fig). The NAD^+^-dependent IDH activity was attributed to ClIDH or to both enzymes.

Interestingly, the glyoxylate cycle probably functions in *C*. *litoralis* KT71 because two key enzymes of this cycle, isocitrate lyase (GenBank accession no. EAQ99477) and malate synthase (GenBank accession no. EAQ99050), together with isocitrate dehydrogenase kinase/phosphatase (GenBank accession no. EAQ98354), are found in its genome. Because of the presence of the complete glyoxylate bypass and of a NADP^+^-specific IDH, *C*. *litoralis* KT71 can use acetate as a carbon source, as was shown by Spring et al. [31]. As two IDHs with different coenzyme specificities reside in *C*. *litoralis* KT71, it will be very interesting to explore the exact physiological functions of these two enzymes. It is known that IDH is a key enzyme in the Krebs cycle that catalyzes the oxidative decarboxylation of isocitrate to yield α-ketoglutarate, and this reaction is coupled to the production of NAD(P)H. NAD^+^-dependent IDH generates NADH to provide electrons for energy production (ATP), and NADP^+^-dependent IDH provides reducing power (NADPH) for biosynthesis, cellular defense against oxidative damage as well as reactive oxygen species (ROS) detoxification. Therefore, it is proposed that the NAD^+^-specific IDH in *C*. *litoralis* KT71 mainly participates in energy metabolism, while the NADP^+^-specific IDH may provide NADPH for biosynthesis during growth on the highly oxidized (energy-poor) compound such as acetate. In addition to *C*. *litoralis* KT71, some other bacteria, such as *Xylella fastidiosa* and *Xanthomonas campestris* (data not shown), also have two IDHs with different coenzyme specificity. Therefore, this phenomenon deserves an in-depth study.

Comparison of the kinetic parameters of ClIDH and NADP^+^-dependent type II IDHs showed that the *K*_m_ value of ClIDH was much higher (262.6±7.4 μM) than those of its NADP^+^-specific counterparts in the type II IDH subfamily, such as those from *Sus scrofa* (5.59 μM) [32], *Rattus norvegicus* (11.5 μM) [33], and *Streptomyces lividans* (2.42 μM) [34]. The decreased affinity of ClIDH towards NAD^+^ resulted in a significant decrease in catalytic efficiency (*k*_cat_/*K*_m_) (0.14 μM^-1^ s^-1^) compared with the homodimeric *S*. *scrofa* NADP-IDH (5.96 μM^-1^ s^-1^) [32] and *R*. *norvegicus* NADP-IDH (9.1 μM^-1^ s^-1^) [33] as well as the monomeric *S*. *lividans* NADP-IDH (9.59 μM^-1^ s^-1^) [34]. Combined with the results from the initial GenBank sequence screen, the low catalytic activity of ClIDH may suggest that this enzyme is old among type II IDHs.

ClIDH showed a *K*_m_ of 937 μM or 90.8 μM for isocitrate in the presence of Mg^2+^ or Mn^2+^, respectively. In addition, the catalytic efficiency (*k*_cat_/*K*_m_) of ClIDH for isocitrate (0.08 μM^-1^ s^-1^) was similar to that of *H*. *thermophilus* NAD-IDH (0.105 μM^-1^ s^-1^) [30] and *S*. *suis* IDH (0.176 μM^-1^ s^-1^) [26].

### Alteration of the coenzyme specificity of ClIDH

The discovery of a type II, NAD^+^-specific ClIDH was primarily based on coenzyme binding hotspot screening, as discussed above. The predicted NAD^+^-specific ClIDH was then confirmed through kinetic analysis. To further examine whether Asp487 and Leu488, which were presumed discriminate NAD^+^ from NADP^+^, truly functioned accordingly, site-directed mutagenesis guided by sequence alignment was performed to convert these two hotspots into Arg and His residues, respectively (Fig 2). Mutant ClIDH (Asp487Arg/Leu488His) was expected to use NADP^+^ as well as NAD^+^ because Arg and His are key amino acid residues for coenzyme discrimination in type II dimeric NADP-IDHs [23–25] (Fig 2).

Kinetic analysis showed that the affinity of the Asp487Arg/Leu488His mutant for NAD^+^ decreased to 40% of that of wild-type ClIDH, as indicated by the observed 2.5-fold increase in the *K*_m_ for NAD^+^ of the mutant enzyme (Table 1). The catalytic efficiency (*k*_cat_/*K*_m_) of the mutant enzyme for NAD^+^ dropped to only 10% of that of wild-type ClIDH (Table 1). However, Asp487Arg/Leu488His showed a remarkable preference for NADP^+^, as indicated by a *K*_m_ value of 156.7±4.8 μM, while the wild-type enzyme was unable to catalyze the reduction of NADP^+^ (Table 1). The mutant enzyme’s catalytic efficiency for NADP^+^ was even higher (0.35 μM^-1^ s^-1^) than that of wild-type ClIDH for NAD^+^ (0.14 μM^-1^ s^-1^). The obtained *k*_cat_/*K*_m_ ratios showed that Asp487Arg/Leu488His presented a 24.3-fold higher preference for NADP^+^ than for NAD^+^, suggesting that the mutant ClIDH was exclusively dependent on NADP^+^ (Table 1). Moreover, the Asp487Arg/Leu488His mutant had a much higher specific activity with NADP^+^ (47 U/mg) than with NAD^+^ (7.5 U/mg). All of these results demonstrate that the coenzyme specificity of ClIDH was successfully switched from NAD^+^ to NADP^+^ by replacing Asp487 and Leu488 in the putative NAD^+^ binding site with the corresponding Arg and His of NADP-IDHs. Thus, the presence of Asp in the binding pockets of type I IDHs and of this novel type II IDH is a hallmark for NAD^+^ specificity. The complete alteration of the coenzyme specificity achieved through replacing of only two amino acids implies that NADP^+^-dependent type II IDHs may have evolved from NAD^+^-dependent ancestors through natural adaptation, similar to what was proposed for the evolution of type I IDHs [10].

### Effects of pH and temperature on ClIDH activity

**Fig 6:**
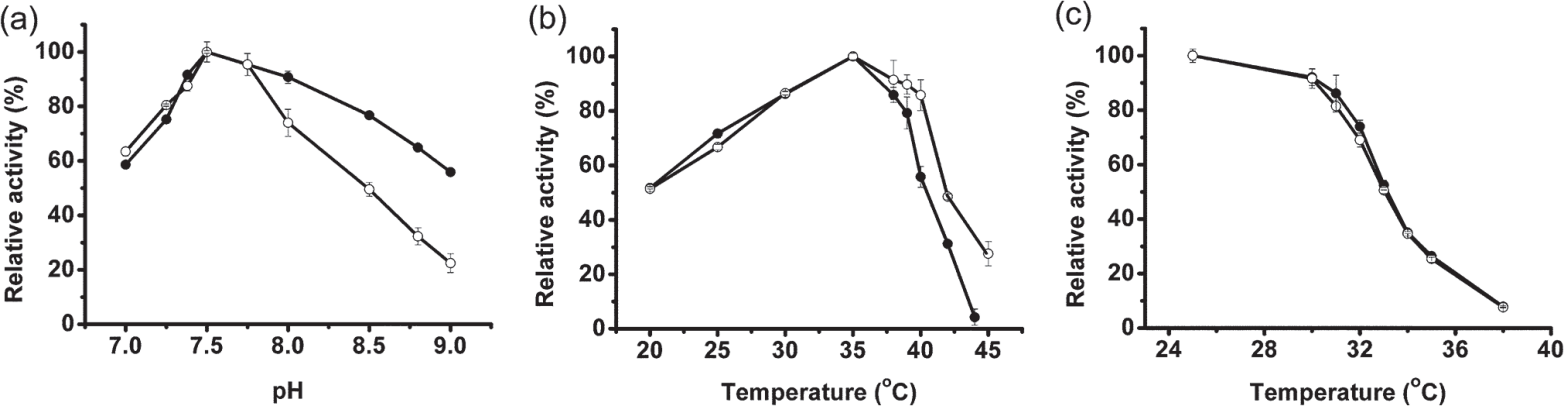
Effects of pH and temperature on the activity of purified recombinant ClIDH. (a) Effects of temperature, from 20°C to 45°C, on enzyme activity in the presence of Mg^2+^ (●) or Mn^2+^ (○). (b) Effects of pH 7.0 to 9.0 on enzyme activity in the presence of Mg^2+^ (●) or Mn^2+^ (○). (c) Heat inactivation profiles of recombinant ClIDH. The activity was measured after 20 min incubation of the enzyme at the temperatures from 25°C to 38°C in the presence of Mg^2+^ (●) or Mn^2+^ (○).

The effects of pH and temperature on the activity of recombinant ClIDH in Tris-HCl buffer were determined. The optimum pH for recombinant ClIDH was 7.5, regardless of whether Mn^2+^ or Mg^2+^ was used as a cofactor (Fig 6A). This value is similar to that of *S*. *suis* NAD^+^-IDH (pH 7.0 with Mn^2+^) [26] but lower than those of *A*. *thiooxidans* NAD^+^-IDH (pH 8.5 with Mg^2+^) [29] and *H*. *thermophilus* NAD^+^-IDH (pH 10.5 with Mg^2+^) [30]. When using Mg^2+^ as a cofactor, the activity of recombinant ClIDH was maintained across a wide pH range. However, in the presence of Mn^2+^, the activity decreased rapidly when at pH above 8.0 and was lost entirely at pH 9.0. The optimum temperature for catalysis was 35°C independently on the metal used for the assay (Fig 6B). This value was slightly higher than that of *S*. *suis* NAD^+^-IDH (30°C with Mn^2+^) [26] but was lower than that of *M*. *capsulatus* NAD^+^-IDH (55°C -60°C with Mg^2+^) [27]. Heat inactivation experiments showed that recombinant ClIDH was not resistant to high temperature; the catalytic activity of the enzyme decreased rapidly after incubation at the temperatures above 30°C, and incubation at 33°C for 20 min caused a 50% loss of activity (Fig 6C). Recombinant ClIDH is less stable at high temperature when compared to other NAD^+^-IDHs such as *S*. *suis* NAD^+^-IDH (stable below 45°C) [26] and *Z*. *mobilis* NAD^+^-IDH (stable below 40°C) [28]. The sensitivity of ClIDH to high temperature may be attributed to its higher-order structure (homohexamer) because the relatively stable *S*. *suis* NAD^+^-IDH and *Z*. *mobilis* NAD^+^-IDH are both compact homodimers [26, 28].

### Effects of metal ions on ClIDH activity

The effects of metal ions on recombinant ClIDH activity were examined (Table 2), and the results showed that the enzyme activity was entirely dependent on the binding of a divalent cation. Mn^2+^ was found to be the most favorable cation for the enzyme, while Mg^2+^ was less favored because recombinant ClIDH had approximately 45.7% of its maximal enzyme activity in the presence of Mg^2+^. Mn^2+^ was also found to be the preferable cation for NAD^+^-IDH from *S*. *suis* [26]. Recombinant ClIDH retained even lower activity in the presence of Co^2+^ (38.3%), Ni^2+^ (1.8%), K^+^ (1.7%) and Na^+^ (1.8%), possibly due to their incapability to interact with the metal ion binding sites of ClIDH. In the presence of Mn^2+^ or Mg^2+^, ClIDH activity was reduced to different levels following the addition of 2 mM Co^2+^, Ca^2+^, or Ni^2+^. Cu^2+^ and Zn^2+^ completely inhibited the activity of recombinant ClIDH; however, monovalent ions such as Na^+^, K^+^, Rb^+^ and Li^+^ had only a moderate effect on ClIDH activity in the presence of Mg^2+^ or Mn^2+^.

**Table 2:** Effects of metal ions on the activity of recombinant ClIDH.

Metal ions	Relative activity (%)	Metal ions	Relative activity (%)	Metal ions	Relative activity (%)
None	2.3±0.4	Mn^2+^	100±2.3^b^	Mg^2+^	100±2^c^
Mn^2+^	100±1^a^	Mn^2+^+Mg^2+^	100.8±3.2	Mg^2+^+Mn^2+^	216.8±3.3
Mg^2+^	45.7±0.9	Mn^2+^+Co^2+^	63±1.6	Mg^2+^+Co^2+^	72.8±0.1
Co^2+^	38.3±1	Mn^2+^+Ca^2+^	70.3±1.3	Mg^2+^+Ca^2+^	17.1±1.4
Ca^2+^	0.8±0.2	Mn^2+^+Zn^2+^	0.8±0.3	Mg^2+^+Zn^2+^	3.1±1
Zn^2+^	0.9±0.2	Mn^2+^+Ni^2+^	20±0.5	Mg^2+^+Ni^2+^	8.8±0.4
Ni^2+^	1.8±0.2	Mn^2+^+Cu^2+^	0	Mg^2+^+Cu^2+^	0
Cu^2+^	0	Mn^2+^+K^+^	101±2.2	Mg^2+^+K^+^	101.2±3.5
K^+^	1.7±1.3	Mn^2+^+Na^+^	103.3±1.8	Mg^2+^+Na^+^	102.7±1.9
Na^+^	1.8±0.4	Mn^2+^+Rb^+^	103.8±2.3	Mg^2+^+Rb^+^	103.8±2.2
Rb^+^	1.5±0.1	Mn^2+^+Li^+^	102.3±0.6	Mg^2+^+Li^+^	103.7±0.5
Li^+^	1.8±0.2				

^a^ A 100% activity corresponds to 61 U/mg protein.

^b^ The activity in the presence of Mn^2+^ alone (61 U/mg protein) is regarded as a 100% value for this column.

^c^ The activity in the presence of Mg^2+^ alone (29 U/mg protein) is regarded as a 100% value for this column.

## Conclusions

The present study reports a novel NAD^+^-specific type II IDH from *C*. *litoralis* KT71 (ClIDH). The phylogenetic position and the ancestral phenotype of ClIDH indicates that ClIDH and other possible NAD^+^-specific type II IDHs may be the ancestors of the modern type II IDHs, which helps to fill an essential gap in the classification of the IDH protein superfamily. The coenzyme specificity of ClIDH can be completely switched from NAD^+^ to NADP^+^ by introducing only two amino acid substitutions, implying that ClIDH has the potential to evolve into an NADP^+^-utilizing enzyme.

## Supporting Information

S1 Fig BLAST analysis revealing 38 putative IDHs of NAD^+^-dependent type II IDH in GenBank using ClIDH as the query. Sequence alignment showed that the coenzyme binding sites of all 38 IDHs included NAD^+^-discriminative Asp and Leu (indicated with stars ★).
(TIF)

S2 Fig Sequence alignment of the second IDH of *C. litoralis* KT71 (ClIDH2, GenBank accession no. EAQ99191), with other typical NADP^+^-IDHs. The NADP^+^ binding sites of *Bacillus subtilis* NADP-IDH (BsIDH) and *Escherichia coli* NADP-IDH (EcIDH) (indicated with stars ★) are completely conserved in ClIDH2, demonstrating the NADP^+^-specificity of ClIDH2. The alignment was performed using ESPript 2.2.
(TIF)
