# Supplementary figures and images for: A Novel Type II NAD+-Specific Isocitrate Dehydrogenase from the Marine Bacterium Congregibacter litoralis KT71
Source: PLoS One. 2015 May 5;10(5):e0125229. doi: 10.1371/journal.pone.0125229 (PMC4420465; doi:10.1371/journal.pone.0125229)

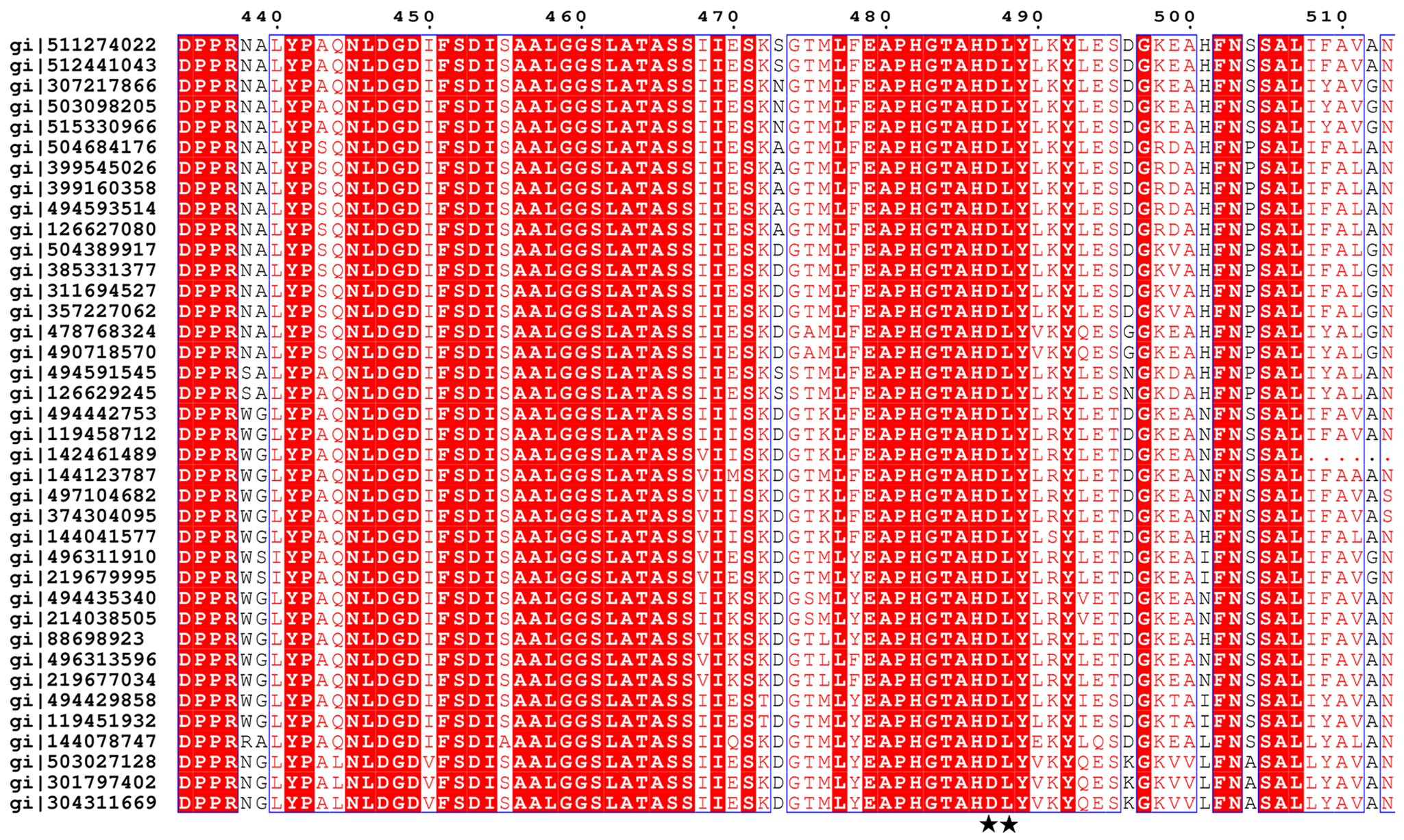

Supplement: S1 Fig — Sequence alignment showed that the coenzyme binding sites of all 38 IDHs included NAD+-discriminative Asp and Leu (indicated with stars ★). (TIF) [file pone.0125229.s001.tif]

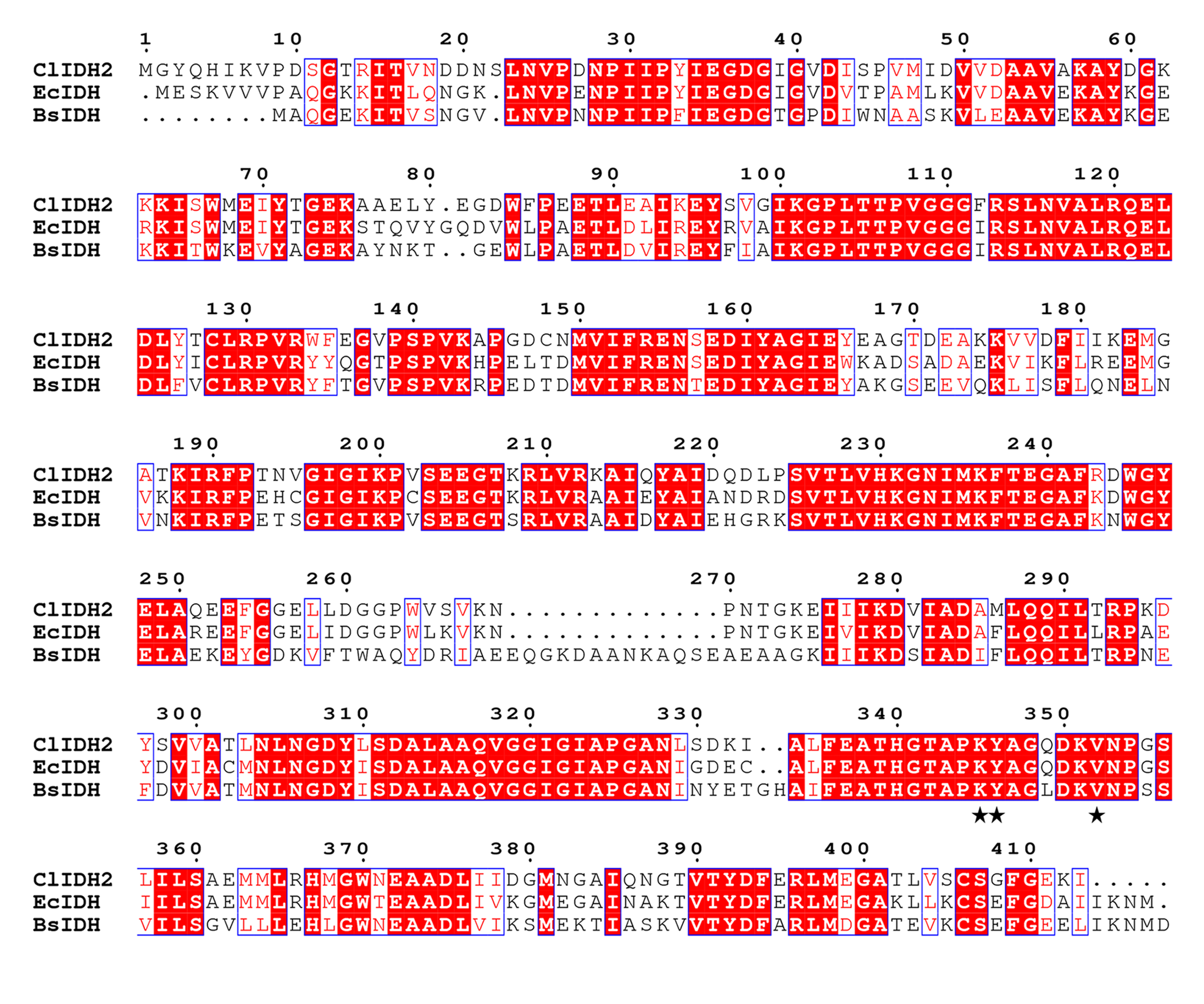

Supplement: S2 Fig — EAQ99191), with other typical NADP+-IDHs. The NADP+ binding sites of Bacillus subtilis NADP-IDH (BsIDH) and Escherichia coli NADP-IDH (EcIDH) (indicated with stars ★) are completely conserved in ClIDH2, demonstrating the NADP+-specificity of ClIDH2. The alignment was performed using ESPript 2.2. (TIF) [file pone.0125229.s002.tif]
